# Gastric neuroendocrine neoplasms and precursor lesions: Case reports and literature review

**DOI:** 10.1097/MD.0000000000028550

**Published:** 2022-01-14

**Authors:** Alina Boeriu, Daniela Dobru, Crina Fofiu, Olga Brusnic, Danusia Onişor, Simona Mocan

**Affiliations:** a Department of Gastroenterology, University of Medicine, Pharmacy, Science, and Technology of Targu Mures, Romania; b Pathology Department, University of Medicine, Pharmacy, Science, and Technology of Targu Mures, Romania.

**Keywords:** enterochromaffin-like-cells, gastric, immunostaining, mitoses, neuroendocrine, proliferative index

## Abstract

**Rationale:**

Gastric neuroendocrine neoplasms (g-NENs) represent a distinctive group of gastric tumors, stratified into different prognostic categories according to different histological characteristics, put forth in the 2018 World Health Organization classification system. The clinical presentations, as well as pathological features, represent important data in establishing the type of the tumor, in estimating the tumor behavior, and in selecting the best therapeutic strategy. In our case series we presented different clinical scenarios that may be encountered in practice regarding gastric NENs. We performed a literature review and discussed diagnostic strategy, current classification system, precursor lesions, and therapeutic options in g-NENs.

**Patient concerns:**

The first patient was a 41-year-old female with weight loss, persistent dyspeptic complaints and a history of pernicious anemia. In the second clinical case a 61-year-old man was admitted with heartburn, abdominal pain, diarrhea and mild iron deficiency anemia. The third patient was a 56-year-old male with a history of neoplasia, admitted for weight loss, dyspeptic complaints, and liver metastases.

**Diagnosis:**

All the 3 patients underwent upper endoscopy with targeted biopsies. Histopathological and laboratory evaluation, together with imagistic evaluation (abdominal ultrasound, endoscopic ultrasound, and magnetic resonance imaging) allowed the distinction between 3 different types of gastric tumors: type 1 enterochromaffin-like-cell G1 NET, type 2 enterochromaffin-like-cell G2 NET, and type 3 G2 NET with liver metastases.

**Interventions:**

Endoscopic polypectomy of the largest lesion was performed in patient with type 1 g-NET and autoimmune chronic atrophic gastritis, followed by regular endoscopic surveillance with biopsies. In type 2 g-NET associated with pancreatic gastrinoma, pancreaticoduodenectomy with total gastrectomy were performed. In type 3 g-NET, detected in metastatic stage, oncologic therapy was performed.

**Outcomes:**

The patients follow-up was selected according to tumor behavior, from regular endoscopic surveillance to oncology follow-up. The prognosis was good in case 1, whilst poorer outcomes were associated with more aggressive tumors in case 2 and case 3.

**Lessons:**

g-NENs are rare tumors with distinct clinical and histological features. Our case series emphasized the role of close collaboration between clinician and pathologist, as well as the importance of a detailed pathology report.

## 1 Introduction

Gastrointestinal neuroendocrine neoplasms (NENs) represent a heterogeneous group of neoplasms originating from endocrine cells that are located in the gastrointestinal tract. These NENs precursor endocrine cells may vary from 1 site to the other, depending on the functional necessities of each site.^[1]^ The incidence of gastrointestinal NENs has increased in recent years and a better patient survival has been reported, mainly related to the improvement of diagnostic techniques, specific immunohistochemical staining methods, and treatment options.^[2–4]^

Gastric NENs (g-NENs) are rare tumors and represent 5% to 23% from all gastrointestinal NENs, according to the published data.^[5–8]^ Although up to 5 neuroendocrine cell types have been described in human gastric mucosa, most g-NENs are composed of nonfunctioning ECL (enterochromaffin-like) cells. These NENs can be preceded by ECL cells hyperplastic and dysplastic lesions, whose oncologic potential has not yet been fully elucidated.^[9]^

Gastric NENs were classified using the 2010 WHO classification of digestive NENs, which consists of a grading system, independently of the immunoprofile of the proliferating cells: grade 1 neuroendocrine tumors (G1 NETs), grade 2 neuroendocrine tumors (G2 NETs), G3 neuroendocrine carcinomas (NECs), and mixed adenoneuroendocrine carcinomas.^[10]^ The classification system was updated in 2017 and 2018, and NENs were divided in neuroendocrine tumors (NETs), NECs, and mixed neuroendocrine–non-neuroendocrine neoplasms (MiNENs).^[11,12]^

In contrast with most of the digestive NENs, gastric tumors may have a specific clinical context, with distinct prognosis and therapeutic management. Thus, 3 types of tumors are recognized: type 1 associated with autoimmune chronic atrophic gastritis (A-CAG), type 2 associated with multiple endocrine neoplasia type 1 (MEN-1) and Zollinger– Ellison syndrome (ZES), and type 3 sporadic.^[12]^

In many cases, tumors remain asymptomatic and may be diagnosed as incidental findings during upper gastrointestinal endoscopy. The classic carcinoid syndrome consisting in cutaneous flushing, tachycardia and secretory diarrhea rarely occurs, but is observed most frequently in patients with liver metastases.^[13]^ The endoscopic appearance of g-NENs consists of polypoid lesions, solitary or multiple. Antral and corporeal biopsies, in addition to biopsies from the tumors, are important to be obtained, in order to determine the type of the tumor. The histopathological report should provide specific data regarding tumor differentiation and proliferation, as well as peritumoral mucosal changes.

Our case reports present clinical scenarios that may be encountered in practice, focusing on clinical and histopathological features of different types of g-NENs with their specific management. Written informed consent was obtained from each patient before enrollment.

## 2 Case reports

### 2.1 Case 1

**Figure 1:**
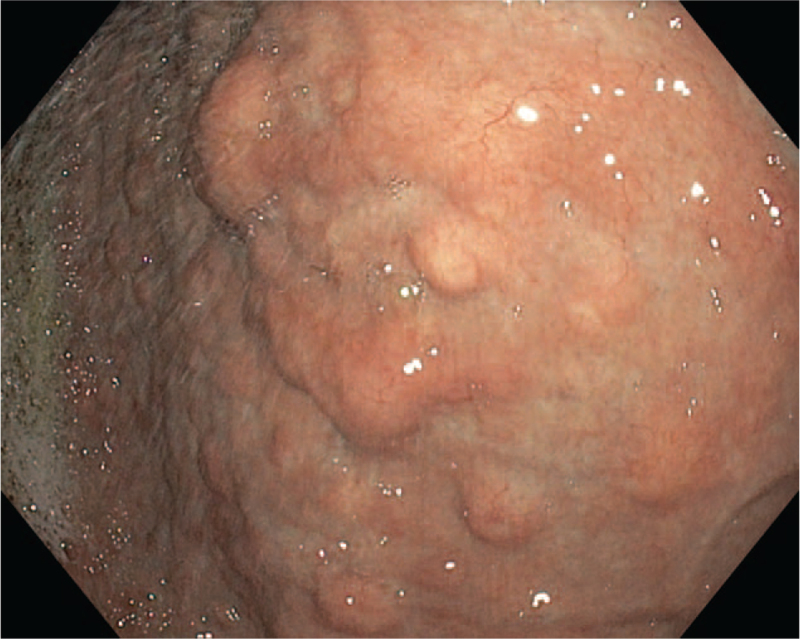
Endoscopic view of multiple polypoid lesions and corporeal atrophic gastritis.

A 41-year-old female with weight loss, persistent dyspeptic complaints and a history of pernicious anemia, underwent upper endoscopy. Corporeal atrophic gastritis with multiple polypoid lesions on the greater curvature and on the anterior wall of the gastric body up to 10 mm in size was identified (Fig. 1). The depth of tumor infiltration was assessed by endoscopic ultrasound. Tumors were limited to the superficial part of the submucosa, without lymph node involvement. Endoscopic polypectomy of the largest tumor (10 mm diameter) was performed, and targeted biopsies from the other polypoid lesions and from the surrounding mucosa were obtained, including distinct fragments from the antrum and corpus. Laboratory evaluation showed elevated fasting serum gastrin levels (1350 pg/mL), as well as serum antibodies to gastric parietal cells.

**Figure 2:**
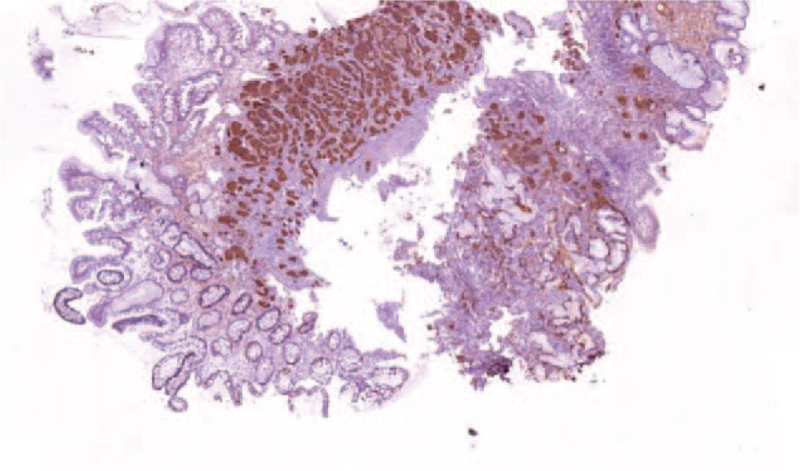
Chromogranin immunostaining revealing a nodular proliferation of positive cells in the mucosa in the setting of extensive intestinal metaplasia and glandular atrophy (×2).

The histopathological evaluation of the polypectomy specimen revealed a nodular tumor of 10 mm diameter, with typical neuroendocrine features invading the mucosa, and with minimal extension in the submucosa. The uniform cells were arranged in nest and showed regular round nuclei, without significant pleomorphism, with only 1 mitoses/10 high-powered fields (HPF). The proliferative index Ki-67 was <2%. The cells were positive for chromogranin A and synaptophysin (Fig. 2). The base of the resected lesion was free of tumor cells. The fragments from the surrounding mucosa displayed histological features corresponding with an autoimmune atrophic gastritis (type A), limited to the corporeal region, with extensive intestinal and pseudopyloric metaplasia. The immunohistochemical examination with chromogranin A and synaptophysin highlighted also a linear and nodular hyperplasia of endocrine cell only in the corpus. The antrum showed minor changes of reactive gastropathy, with no inflammation, intestinal metaplasia, glandular atrophy, or neuroendocrine cell hyperplasia. No *Helicobacter pylori* was identified in the specimens.

Based on clinical circumstances and morphological features, the diagnosis of type 1 ECL cell NET G1 associated with A-CAG was established, and regular endoscopic surveillance was recommended.

### 2.2 Case 2

**Figure 3:**
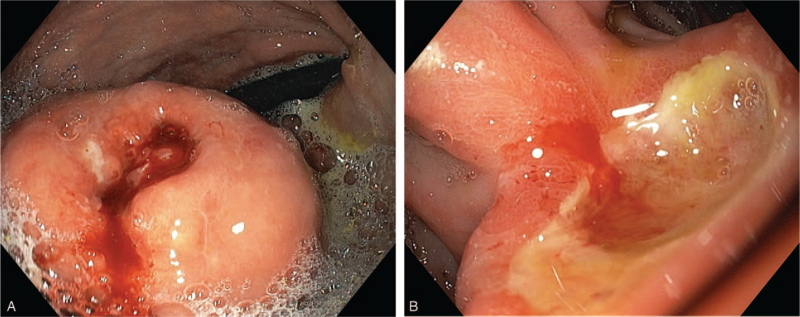
Endoscopic view of tumor with central ulceration in the gastric body (A) and a large duodenal ulcer (B).

A 61-year-old male with a history of peptic ulcer disease underwent upper gastrointestinal endoscopy for heartburn, abdominal pain, diarrhea, and mild iron deficiency anemia (hemoglobin = 11.82 g/dL). Laboratory showed elevated fasting serum gastrin levels (969 pg/mL). Serum parathormon, calcium and prolactin levels were normal. At the level of gastric body multiple polypoid lesions up to 15 mm in size were detected, and 1 large protrusive lesion (4 cm in size) with central ulceration. At the level of duodenum, multiple ulcers were found (Fig. 3A and B). Biopsies were obtained from the largest gastric tumor and the surrounding mucosa.

Microscopic examination of the biopsy fragments revealed a neuroendocrine type proliferation, without significant nuclear pleomorphism or necrosis. The tumor was extended through muscularis mucosae in the submucosa. The cells were positive for chromogranin A and synaptophysin. The surrounding mucosa was hypertrophic, with different type of ECL cell proliferation, including linear, nodular and dysplastic lesions in the antrum as well as in the corpus, without metaplastic changes or glandular atrophy. The inflammation was insignificant, and no *H pylori* was identified. The foveolar component and the glandular component in both gastric regions showed a hyperplastic appearance.

In the setting of hypergastrinemia, duodenal ulcers, neuroendocrine gastric tumors and hypertrophic gastric mucosa, we suspected a Zollinger–Ellison syndrome. Magnetic resonance imaging was performed to identify the primary tumor site. A 16/13 mm lesion was detected at the level of the head of the pancreas, in close contact with duodenal wall. Magnetic resonance imaging also revealed corporeal gastric tumor of 46/52 mm in size, and enlarged lymph nodes measuring 12 mm.

In this case of type 2 ECL cell NET the surgical treatment was recommended, and pancreaticoduodenectomy with total gastrectomy were performed. Macroscopic evaluation of the resected specimen showed multiple nodular lesions in the stomach, protruding from the mucosa, with dimensions between 10 and 60 mm, distributed in the gastric body. The largest polypoid lesion was ulcerated on the surface. In the perigastric adipose tissue 16 lymph nodes were found, the largest with 10 mm diameter. In the duodenum, multiple ulcers were identified, with smooth margins, penetrating the duodenal wall, reaching 15 mm in size. In the head of the pancreas, a small nodule, well-demarcated, grey tan, of 25 mm in size, was identified. Twelve lymph nodes were found in the peripancreatic adipose tissue.

**Figure 4:**
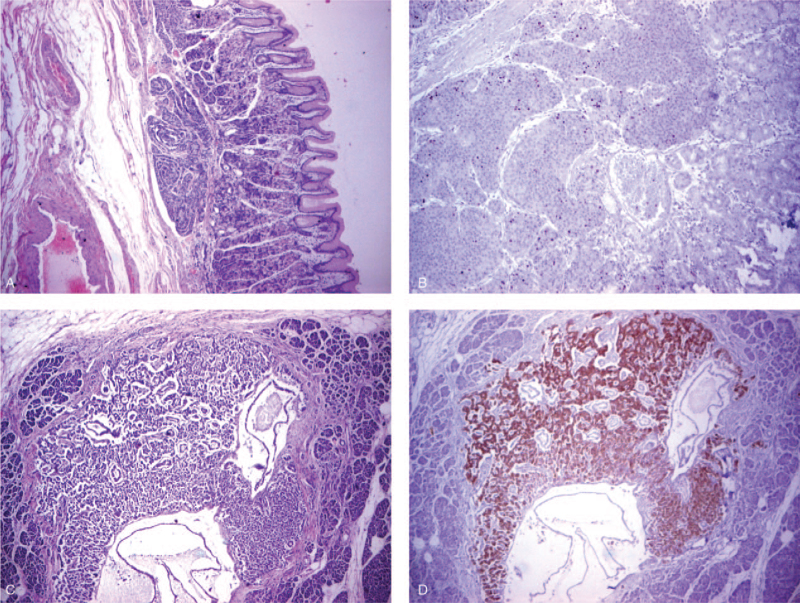
Histological assessment of gastric and pancreatic lesions: (A) nodule of uniform neuroendocrine cells in the fundic type mucosa, with no intestinal metaplasia or glandular atrophy (HE, ×2); (B) immunohistochemical staining for Ki-67 of the gastric nodule (×2); (C) pancreatic tumor, well circumscribed, with a nested pattern (HE, ×2); (D) pancreatic tumor positive for gastrin (×2). HE = hematoxylin–eosin stain.

Microscopic examination of the largest gastric tumor revealed a neuroendocrine neoplasm invading the mucosa and submucosa, reaching the muscularis propria. The cells were uniform, with abundant, focally eosinophilic cytoplasm, regular round nuclei, small nucleoli, with 11 mitoses/10 HPF, and Ki-67 index of 3%. No embolies were identified and necrosis was not present. The pancreatic tumor was a well-differentiated NET, with uniform cells, without nuclear pleomorphism, with Ki-67 proliferative index <2%, and 1 mitosis/10 HPF. The cells were positive for chromogranin A, synaptophysin, gastrin and cluster of differentiation 56 (Fig. 4). No metastases were detected in the 12 peripancreatic and 16 perigastric lymph nodes. Thus, histopathologic examination of the resected specimen confirmed the presence of a gastrin producing neuroendocrine G1 tumor in the head of the pancreas. The largest gastric tumor represented a G2 NET, infiltrating the gastric submucosa. The smaller gastric nodules were G1 NETs, with Ki-67 index <2%, and 1 mitosis/10 HPF.

### 2.3 Case 3

**Figure 5:**
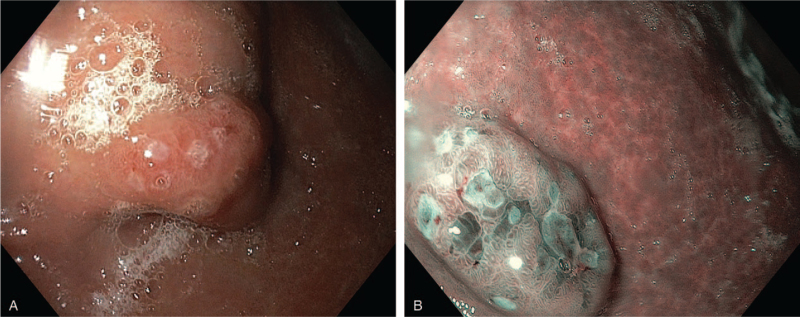
Endoscopic view of antral tumor (A) showing irregular pattern on NBI (B).

A 56-year-old male patient with a personal history of prostatic adenocarcinoma successfully treated by surgical therapy was referred for weight loss, dyspeptic complaints and multiple liver metastases detected on abdominal ultrasound. We performed an upper gastrointestinal endoscopy with narrow-band imaging. A 2 cm polypoid lesion was detected in the antrum showing an irregular mucosal pattern on narrow-band imaging (Fig. 5), while surrounding antral and corporeal mucosa showed normal mucosal and vascular patterns. Biopsies were performed from the antral lesion, from the surrounding antral mucosa and from the corporeal mucosa.

**Figure 6:**
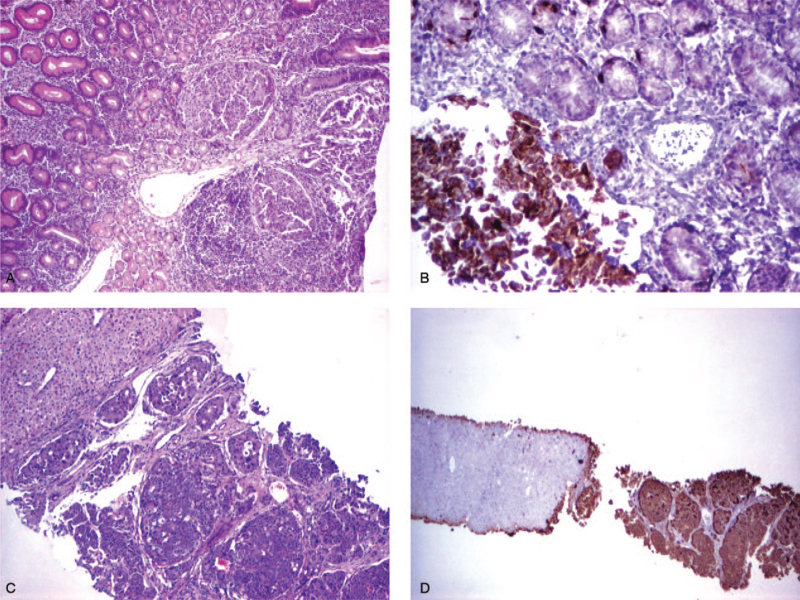
Histological assessment of gastric tumor and liver metastasis: (A) large nodules of uniform cells with neuroendocrine features in the antral mucosa (HE, ×4); (B) positive cells for chromogranin; (C) liver biopsy with large nodules of neuroendocrine cells, with pseudoglandular architecture (HE, ×4); (D) liver biopsy with positive cells for chromogranin (×2). HE = hematoxylin–eosin stain, NBI = narrow-band imaging.

The histopathological evaluation of the biopsies demonstrated a proliferation of small, uniform cells with solid, trabecular and pseudoglandular architecture, consisting with a neuroendocrine tumor (Fig. 6A). Four mitoses per 10 HPF were detected, and Ki-67 index was 5%, corresponding with G2 NET. Immunohistochemical profile showed positive staining with chromogranin A (Fig. 6B), synaptophysin, cluster of differentiation X2 (corresponding with a primary gastric lesion), and negative staining with cytokeratin 7 and 20, and for prostate-specific antigen. *H pylori*, associated with active inflammation and regenerative foveolar hyperplasia were detected in nearby gastric mucosa, without evidences of glandular atrophy or neuroendocrine cell hyperplasia.

Ultrasound-guided liver biopsy confirmed liver metastases from the neuroendocrine tumor, with positive synaptophysin and chromogranin on immunohistochemistry (Fig. 6C and D). In these circumstances, we concluded that the tumor represented a type 3 gastric NET, detected in an advanced, metastatic stage. The patient did not consent for surgical treatment of the tumor, and oncologic therapy was indicated.

## 3 Discussion

The gastrointestinal tract has different types of endocrine cells that vary from 1 site to another, depending of the functional needs of each region. Considering the diversity of the endocrine population of cells and the hormonal complexity of the gastrointestinal system, it is surprising that the diseases of the system are so limited. Most of the lesions are represented by tumors.^[14]^

In the stomach, endocrine cells have an essential role in acid secretion. The antrum contains gastrin secreting cells or G-cells. G-cells are located at the base of mucous neck cells and in the superficial mucous gland cells. Gastrin represents a direct stimulus for parietal cells to produce acid, and also acts like a direct proliferative stimulus on ECL cells. ECL cells are found diffusely throughout the glands in the body of the stomach.

ECL cells proliferation may occur in patients with corporeal atrophic gastritis, in patients with MEN-1 syndrome, or in patients with hypo/aclorhydria induced by long-standing proton pump inhibitor therapy.^[15]^ Peritumoral mucosa in both type 1 and type 2 g-NETs shows hyperplastic and/or dysplastic proliferations of ECL cells, which are regarded as precursor lesions for these NETs.^[16]^

Some research focused on risk related to ECL cells changes, but it is difficult to define which type of proliferation has the greatest potential for neoplastic transformation.^[16]^ Vanoli et al^[17]^ demonstrated that severe ECL cells hyperplasia consisting in more than 6 chains of linear hyperplasia per mm, as well as ECL cell dysplasia, poses an increased risk for neuroendocrine tumor development in patients with type A-CAG.^[18]^ Severe linear hyperplasia represents a predictive factor for type 2 gastric NET development in patients with MEN-1 syndrome, according to data reported by Berna et al.^[19]^

Although ECL cells are not readily recognized on routine hematoxylin and eosin staining, they contain vesicular granules highlighted with immunohistochemical staining. Immunohistochemical markers of endocrine differentiation are used to highlight normal and neoplastic cells, and they can be divided into 4 classes: cytosolic or cell membrane markers (most common neuron specific enolase or NSE and more recently vesicular monoamine transporter-2), small vesicle associated markers (most common synaptophysin), secretory granule associated (including chromogranin A), and specific peptide hormone markers (such as serotonin, somatostatin, and gastrin).^[20,21]^ Each marker has a different specificity and sensitivity. Therefore, a negative or positive reaction with a single marker cannot be recommended in routine practice to establish or exclude the diagnosis of an endocrine tumor.^[14]^

NETs classification has been a highly debated subject over the years. WHO classification of digestive NENs was adopted in 2010. Four types of g-NENs have been described, based on the histopathological assessment of the number of mitoses per 10 HPF and the proliferative activity (Ki-67 index). G1 NETs are tumors presenting <2 mitoses/10 HPF, with a Ki-67 index <2%. G2 NETs present between 2 to 20 mitoses/10 HPF, and a Ki-67 index between 3% and 20%. Tumors characterized by more than 20 mitoses/10 HPF, and a Ki-67 proliferative index >20% represent NECs.^[10]^

In 2017, a new WHO classification divided NENs in 3 types of well differentiated NETs (G1 NETs present <2 mitoses/10 HPF, Ki-67 index <3%; G2 NETs present 2 to 20 mitoses/10 HPF, and a Ki-67 index between 3% and 20%; grade 3 neuroendocrine tumors (G3 NETs) present more than 20 mitoses/10 HPF, and a Ki-67 proliferative index >20%), and 2 types of poorly differentiated NECs (small-cell type and large-cell type, with more than 20 mitoses/10 HPF, and a Ki-67 proliferative index >20%). MiNENs represent a distinct category, combining neuroendocrine and non-neuroendocrine components.^[11]^

The most recent WHO classification system^[12]^ divided g-NENs in well differentiated NETs, poorly differentiated NECs (small cell and large cell), and MiNENs. Distinct subtypes of NETs are defined: histamine-producing ECL cell NET (Type 1 and Type 2); Type 3 NET (G1 NET, G2 NET, and G3 NET); somatostatin-producing D-cell NET; gastrin-producing G-cell NET; serotonin-producing enterochromaffin-cell NET.^[22]^

Type 1 ECL cell NETs represent 70% to 80% of all GNETs and occur in patients with type A-CAG.^[23,24]^ These are related to hypergastrinemia due to a compensatory hyperplasia of antral G cells, in response to hypo/achlorhydria induced by the loss of specialized glands in the body. Hypergastrinemia represents the stimulus for hyperplastic proliferation of ECL cells and the development of NETs. Tumors develop as multiple polypoid lesions, usually small (<10 mm), in the corpus of the stomach or in the gastric fundus.^[25,26]^ The histological evaluation shows well-differentiated cells, growing in trabecular patterns, usually confined to mucosa or submucosa, immunoreactive for chromogranin A and synaptophysin, vesicular monoamine transporter 2, and somatostatin receptor 2A.^[25,27]^ Tumors are classified as G1 NETs, with proliferation marker (Ki-67 index) less than 2%. Biopsies from surrounding mucosa show atrophic gastritis and hyperplasia of ECL cells. These proliferative lesions are considered precursors of NETs.

The behavior of type 1 g-NETs is typically indolent, although a few cases of aggressive tumors (G3 NET) have been described.^[28–30]^ The possibility of vascular invasion and metastases endorses the role of endoscopic ultrasonography in the assessment of the depth of tumoral invasion and lymph nodes involvement, especially in tumors greater than 10 to 20 mm in size.^[31,32]^

Type 2 ECL cell NETs represent 5% to 6% of all g-NETs and occur in patients with hypergastrinemia, secondary to ZES and MEN-1 syndrome.^[24]^ The diagnostic work-up should comprise a screening for possible associated parathyroid and pituitary tumors, as well as the assessment of parathyroid hormone level, ionized calcium, and plasma prolactin.^[33–35]^ The endoscopic appearance of gastric tumors is similar with type 1 g-NETs, consisting of multiple small nodular lesions in the gastric body and fundus, but the surrounding mucosa is hypertrophic and various types of hyperplastic ECL cells proliferation can be found. Type 2 NETs are also well-differentiated tumors, confined to mucosa and submucosa in the majority of cases. Most of them are G1 NETs, rarely G2 NETs. Metastases may occur in 10% to 30% of patients.^[36]^

Type 3 NETs represent 15% to 20% of all g-NETs.^[24]^ There are solitary and large tumors (>2 cm) arising in any part of the stomach, most frequently in males over 50 years old, unrelated to gastrin levels. Tumors occur in normal (nonatrophic) mucosa, without ECL cells proliferations. They may display different proliferation degrees (G1, G2, or G3).^[37]^ Aggressive tumors can infiltrate the muscularis propria with angio-invasion, lymph node, and liver metastases.^[26]^

A distinct rare type of g-NEN was described as type 4 g, usually occurring in men over 60 years old. Tumor has non-ECL origin and is not associated with autoimmune gastritis or gastrinoma, being gastrin-independent. Tumor is large (>4 cm), located anywhere in the stomach,^[8,15,38]^ showing positive immunostaining with synaptophysin and cytosol markers NSE and PGP9.5, while chromogranin A is absent or focally expressed.^[16,39]^ According to the recent WHO classification system, tumor is a NEC, with aggressive behaviour, vascular invasion, and metastases.^[40]^

Regarding the therapeutic approach, annual or twice yearly endoscopic surveillance for small tumors (<10 mm diameter), surgical therapy (antrectomy, gastrectomy), endoscopic therapy (polypectomy, endoscopic mucosal resection or endoscopic submucosal dissection) for tumors >10 mm diameter, long-acting somatostatin analogs lanreotide and octreotide, are available options for type 1 g-NETs.^[41–47]^ A gastrin/cholecystokinin 2 receptor antagonist, Netazepide, showed promising results, by decreasing the number and the size of the tumors and by normalizing CgA levels.^[48]^

Some authors advocate antrectomy in order to eliminate the gastrin stimulus that promotes tumor growth, and local resection of the largest tumors with subsequent endoscopic surveillance of the gastric remnant.^[15,41,42]^ Vanoli et al reported in 1 patient with type A-CAG and type I gastric NET treated by antrectomy a decrease in gastrin levels to undetectable levels, and a regression of ECL cell hyperplasia postoperatively, without NET recurrence. However, in another patient treated by antrectomy, postoperatively gastrin levels remained higher than normal, and NET recurrence was reported 5 years after the antrectomy. A possible explanation for this behavior could be the persistence of “ectopic” gastrin cells in atrophic corporeal mucosa and hypergastrinemia which promotes tumor growth.^[17]^ In our patient with CAG and type 1 g-NET, endoscopic resection of the largest tumor and endoscopic surveillance were recommended (case 1).

Surgical treatment is recommended for patients with type 2 g-NETs, primarily directed to underlying disease (removal of gastrinomas, in order to reduce the ECL cells stimulation).^[49]^ In the presence of metastases, cytoreduction surgery is recommended to control symptoms and hormonal hypersecretion.^[50]^ Octreotide showed good results in tumors regression in ZES or MEN-1.^[51]^ Pancreaticoduodenectomy with total gastrectomy were performed in patient with type 2 gastric NET (case 2).

Solitary type 3 g-NETs arising in normal mucosa may have an aggressive behavior, requiring a radical surgical therapy. Endoscopic treatment (endoscopic mucosal resection or endoscopic submucosal dissection) was indicated in small (<20 mm diameters) G1 or G2 tumors, with no lymph node or distant metastases.^[52]^ In the case of metastatic liver disease, surgery, somatostatin analogues, chemotherapy (streptozocin, 5-fluorouracil with leucovorin, cyclophosphamide, doxorubicin, oxaplatin, dacarbazine), and locoregional control methods (targeted radionucleotide therapies, transarterial chemoembolization, radiofrequency ablation) are recommended.^[33]^

The patient with antral tumor and liver metastases (case 3) declined surgical treatment and chose the medical therapy. The unique aspect of this case consists of the presence of a previously treated prostatic malignancy, with an apparently good outcome in the absence of lymph nodes and bone metastasis, and the subsequent detection of an advanced metastatic disease. Positive immunohistochemical staining for chromogranin proved neuroendocrine nature of gastric tumor and liver metastases. After a literature search, we found that liver metastases secondary to prostatic adenocarcinoma are very uncommon, and usually occur in patients presenting a systemic aggressive disease with bone and/or lymph node metastases.^[53,54]^ As concern the gastric tumor, there have been reported few cases of prostate adenocarcinoma metastatic to the stomach. In such cases, immunostaining positive for prostate-specific antigen and cytokeratin, and negative for chromogranin suggests the diagnosis.^[55,56]^ Another particularity of this case is the detection of a well-differentiated G2 tumor with liver metastases. Rare cases of G 2 type 3 NETs (solitary, developed in the absence of hypergastrinemia) with liver metastasis or ovarian metastasis were previously reported.^[57,58]^

## 4 Conclusions

g-NENs are rare tumors with distinct clinical and histological features. The histological diagnosis and tumor grading according to standard terminology is important in estimating tumor behavior and in adopting the best therapeutic decision. Close communication between the histopathologist and clinician is required, with an analysis of the relevant clinical data, correlated with histologic analysis of tumor and nontumor tissue. Given the oncogenic potential of ECL cells changes, a regular endoscopic and histological follow-up of the patient is advisable when ECL cells hyperplastic and dysplastic proliferations are detected in gastric biopsy specimens.

## Author contributions

**Conceptualization:** Alina Boeriu.

**Investigation:** Simona Mocan.

**Methodology:** Danusia Onişor.

**Validation:** Daniela Dobru.

**Writing – original draft:** Alina Boeriu, Crina Fofiu, Olga Brusnic.
